# Quantum Transport Enhancement by Time-Reversal Symmetry Breaking

**DOI:** 10.1038/srep02361

**Published:** 2013-08-06

**Authors:** Zoltán Zimborás, Mauro Faccin, Zoltán Kádár, James D. Whitfield, Ben P. Lanyon, Jacob Biamonte

**Affiliations:** 1 Institute for Scientific Interchange, Via Alassio 11/c, 10126 Torino, Italy; 2 Department of Theoretical Physics, University of the Basque Country UPV/EHU, P.O. Box 644, E-48080 Bilbao, Spain; 3 Vienna Center For Quantum Science and Technology, Boltzmanngasse 5 1090 Vienna, Austria; 4 Institut für Quantenoptik und Quanteninformation, Otto-Hittmair-Platz 21a 6020 Innsbruck, Austria; 5 Centre for Quantum Technologies, National University of Singapore, Block S15, 3 Science Drive 2, Singapore 117543

## Abstract

Quantum mechanics still provides new unexpected effects when considering the transport of energy and information. Models of continuous time quantum walks, which implicitly use time-reversal symmetric Hamiltonians, have been intensely used to investigate the effectiveness of transport. Here we show how breaking time-reversal symmetry of the unitary dynamics in this model can enable directional control, enhancement, and suppression of quantum transport. Examples ranging from exciton transport to complex networks are presented. This opens new prospects for more efficient methods to transport energy and information.

Understanding quantum transport is key to developing more robust communication networks, more effective energy transmission, and improved information processing devices. Continuous time quantum walks have become a standard model to study and understand quantum transport phenomena^1^,^2^,^3^,^4^,^5^,^6^. Time-reversal symmetric (TRS) Hamiltonians have characterized all quantum walk models to date. This symmetry implies that the site-to-site transfer probability at time *t* = *T* is the same as at time *t* = −*T*, thereby prohibiting directional biasing. Here we introduce and study continuous time “chiral” quantum walks whose dynamics break TRS. Our findings show that the breaking of TRS offers the possibility of directional biasing in the unitary dynamics and allows one to suppress or enhance transport relative to the standard quantum walk. One subtlety of this effect is that time-reversal asymmetry cannot affect the site-to-site transport in some simple cases, such as linear chains and trees – this is proven in the Methods Section. Prior efforts in the area of quantum transport have focused on controlling and directing transport using either *in situ* tunable Hamiltonians^7^,^8^,^9^ or tailoring specific initial states^10^. In contrast to known approaches, we consider states initially prepared in the standard *site-basis* and time-independent Hamiltonians that induce time-asymmetric evolutions in the unitary part of their dynamics.

While the effect of TRS breaking dynamics in the context of quantum walks has not been investigated, it has been studied intensely in the condensed matter literature. These investigations range from the very early work of Peierls^11^, through the famous examples of the Hofstadter butterfly^12^ and the Quantum Hall^13^ effect, up to recent research on TRS breaking in topological insulators^14^ and on artificial gauge fields in optical lattice potentials^15^. In contrast to the present study, these works always concentrated on many-body dynamics in regular lattices, while in the context of quantum walks, one is instead interested in characteristically different scenarios: e.g. the dynamics of single individual particles or excitons (usually starting from a single site) moving on complicated networks (sometimes with a bath included). The examples we study are from a variety of modern research topics (e.g. photosynthetic exciton transport and complex networks) and considerably extend the domain of application of known results about TRS breaking beyond solid state applications.

To demonstrate the effect of TRS breaking, we chose five examples which illustrate the main ideas of directionality, suppression and enhancement of transport. The first example is a unitary quantum switch where the phase, that is the time reversal asymmetry parameter, controls the direction of quantum transport. The second example examines transport in a linear chain of triangles, showing a 633% transport speed-up for the chiral quantum walk. In connection with this, we also demonstrate complete suppression of chiral quantum walks on loops with an even number of sites. We then consider a system widely studied in the exciton transport literature: the Fenna-Matthew-Olsen complex (FMO). Although this naturally occurring system is highly efficient, we find that the introduction of chiral terms allows for an enhancement of transport speed by 7.68%. It has recently been shown that the effect does appear in similar light harvesting complexes^16^. Finally, to investigate the robustness of the effect of TRS breaking on transport, we consider randomly generated small-world networks. By appending time-reversal asymmetric terms to only the edges of the network connected to the final site, we could increase the speed of the site-to-site transport on these randomly generated graphs significantly, up to 130%.

## Results

In the standard literature on continuous time quantum walks^1^,^2^,^3^,^4^,^5^, the time-independent walk Hamiltonian is defined by a real weighted adjacency matrix *J* of an underlying undirected graph, 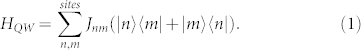
The condition that the hopping weights *J_nm_* are real numbers implies that the induced transitions between two sites are symmetric under time inversion. We can break this symmetry while maintaining the hermitian property of the operator by appending a complex phase to an edge: 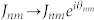 resulting in a continuous time *chiral quantum walk* (CQW) governed by 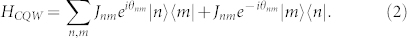
When acting on the single exciton subspace, the Hamiltonian given in Eq. (2) can be expressed in terms of the spin-half Pauli matrices: 
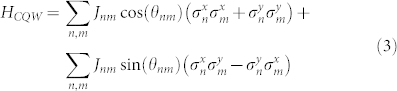
which arises in a variety of physical systems when magnetic fields are considered. We explore a proof-of-concept experimental demonstration of this effect in Supplementary Information, Section S2.

In the CQW framework, we investigate coherent quantum dynamics and incoherent dynamics within the Markov approximation. Both types of evolution are included in the Lindblad equation^17^,^18^,^19^,^20^: 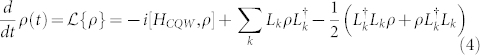
where ρ(*t*) is the density operator describing the state of the system at time *t* and *L_k_* are Lindblad operators inducing stochastic jumps between quantum states. For example, using the usual terminology of Markovian processes, we call site *t* a trap if it is coupled to site *s* by the Lindblad jump operators, *L_k_* = |*t*〉〈*s*|. The site-to-site transfer probability, *P_n_*_→*m*_(*t*) = 〈*m*|ρ(*t*)|*m*〉, gives the occupancy probability of site *m* at time *t* with initial condition ρ(0) = |*n*〉〈*n*|. Note that the present study, while utilizing open system dynamics, is not related to the enhancement of transport due to quantum noise^21^,^22^ which has been well studied in the context of photosynthesis^22^,^23^. Here the emphasis is instead on the effect the breaking time-reversal symmetry of the Hamiltonian dynamics can have on transport.

To quantify the transport properties of quantum walks, we use the *half-arrival time*, *τ*_1/2_, as the earliest time when the occupancy probability of the target site is one half. We will also make use of the transport speed, *ν*_1/2_, defined as the reciprocal of *τ*_1/2_.

**Figure 1:**
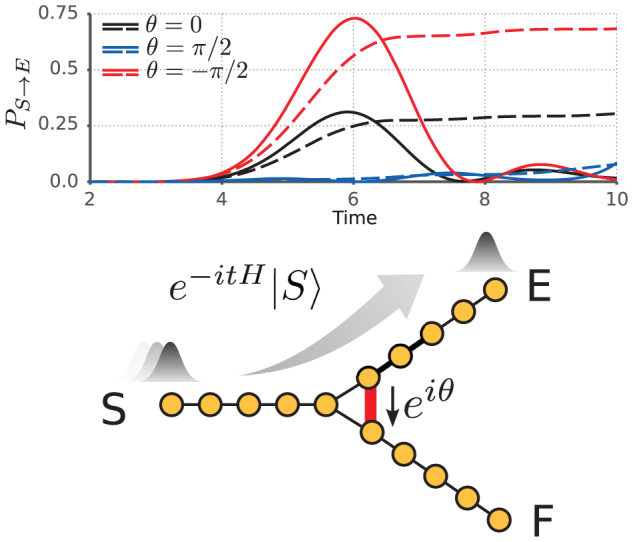
The quantum switch. (a) Directional biasing: enhanced transport in the preferred direction. (b) The plot shows the occupancy probability *P_S→E_* of site *E* with the particle initially starting from site *S* with and without a sink (dashed and solid lines, respectively). This evolution is time-reversal asymmetric as replacing *t* with −*t* results in the particle moving from site *S* towards site *F*. When starting at site *E*, the particle evolves towards site *F*. By replacing *t* with −*t*, a particle initially at site *E* evolves towards the initial configuration (b). To recover time-reversal symmetric transition probabilities in the evolution (b), requires that one also performs the antiunitary operation^24^ on the Hamiltonian mapping *θ* to −*θ*. This has the same effect as reflecting the configuration horizontally across the page while leaving the site labels intact.

We now introduce a quantum switch which enables directed transport and could, in principle, be used to create a logic gate and offer future implementations of transport devices to store and process energy and information. Figure 1 presents an example of this switch. The value of a phase (*e*^*iθ*^) appended to a single control edge across the junction allows selective biasing of transport through the switch. The maximal biasing occurs at |*θ*| = *π*/2, and the sign determines the direction. The first maxima of *P_S_*_→*E*_(*t*) (transfer probability from site S to E) in the unitary dynamics without traps can be enhanced by 134% or suppressed to 91% with respect to the non-chiral case. When considering traps in the Lindbladian evolution, the optimal transport efficiency is 81.4% in the preferred direction. The switch violates TRS as *P_S_*_→*E*_(−*t*) ≠ *P_S_*_→*E*_(*t*). By using *P_S_*_→*E*_(−*t*) = *P_E_*_→*S*_(*t*) and the symmetry of the configuration *P_E_*_→*S*_(*t*) = *P_S_*_→*F*_(*t*), we conclude that transport is biased towards the opposite pole when running backwards in time, see Figure 1 Note that the behaviour of the switch is largely independent of the length of the connecting wires.

**Figure 2:**
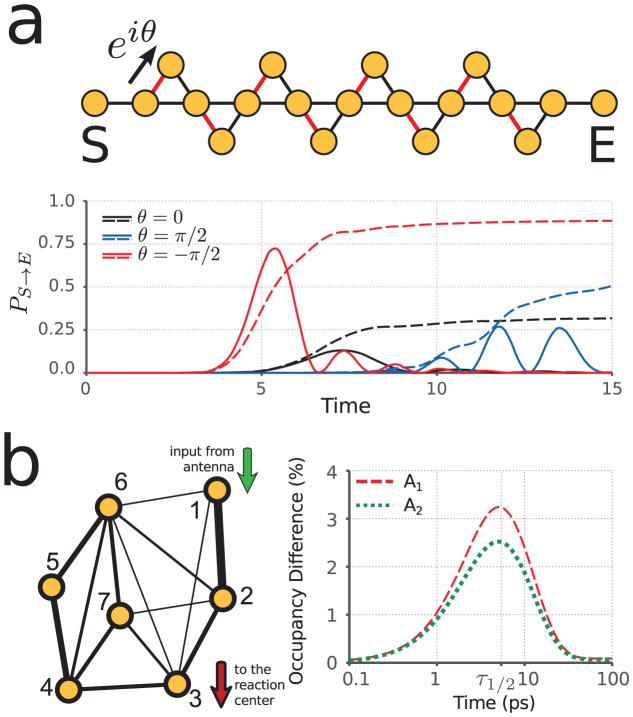
(a) Triangle chain and (b) the FMO complex. (a) The phase *e*^*iθ*^ is applied to the red edges simultaneously in the triangle chain. The plot illustrates the occupancy probability at the end site *E* as a function of time for different values of the phase *θ* with and without trapping (dashed and solid lines, respectively). (b) shows the occupancy difference with respect to the time reversal symmetric Hamiltonian of the FMO complex. We use an optimization procedure to enhance the transport. While holding the magnitude of the couplings constant, we optimize two sets of phases, *A*_1_ and *A*_2_, which correspond to seven and three edges with an enhancement at *τ*_1/2_ of 3.25% and 2.25%, respectively.

We will now utilize the directional biasing of the triangle to give an example of a speed-up of chiral walks. Using the composition of eight triangular switches as depicted in Figure 2a, by simultaneously varying all phases along the red control edges to the same value, we examine the effect of time-reversal asymmetry on transport. We find that the occupation probability as a function of *θ* is symmetric about ±*π*/2 with the negative value corresponding to maximal enhancement and the positive value to maximal suppression. Unlike the occupation probability maxima in the switch, here the first apexes are separated in time. When we include trapping, the half-arrival time is reduced from the non-chiral value *τ*_1/2_ = 38.1 to 5.2 which represents a 633% enhancement. To conclude this section we focus on suppression of transport by chiral quantum walks. A good example is the polygon with an even number of sites. In this case, complete suppression can be achieved by appending a phase of *π* to one of the links in the cycle; thereby rendering it impossible for the quantum walker to move to the diametrically opposite site. This is a discrete space version of a known effect in Aharonov-Bohm loops^25^. The proof that the site-to-site transfer probability is zero in this case for all times also in our discrete-space and open-system walks can be found in the Methods Section. However, note that the discrete even-odd effect, which implies that only loops comprised of odd particles can exhibit transport enhancement, and only even loops may exhibit complete suppression, has no known continuous analog.

In natural and synthetic excitonic networks such as photosynthetic complexes and solar cells, we are faced with non-unitary quantum evolution due to dissipative and decoherent interaction with the environment. Studies have shown that dissipative quantum evolution surpasses both classical and purely quantum transport (for interesting recent examples see^20^,^21^). A widely studied process of such dissipative exciton transport is the one occurring in the Fenna-Matthews-Olsen complex (FMO), which connects the photosynthetic antenna to a reaction centre in green sulphur bacteria^22^,^27^,^28^,^29^. Due to the low light exposure of these bacteria, there is evolutionary pressure to optimize exciton transport. Therefore, the site energies and site-to-site couplings in the system are evolutionarily optimized, yielding a highly efficient transport^23^. However, it is an open question whether or not there occurs time-reversal asymmetric hoping terms in these systems, and whether these are optimized. Recent 2D Electronic Spectroscopy results lead to the conclusion that, e.g., in the light harvesting complex LH2 hopping terms with complex phases are indeed present^16^. Here we ask whether such TRS breaking interactions may further enhance the efficiency of the light harvesting process. We consider the traditional real-hopping Hamiltonian modeling transport on the FMO, and allow for TRS breaking by introducing complex phases and find that the transport speed can be further increased. We study the seven site model of the FMO using an open system description that includes the thermal bath, trapping at the reaction centre, and recombination of the exciton^22^,^26^,^27^. By performing a standard optimization procedure (as outlined in the Supplementary Information, Section S3) that varies the phase on a subset of seven edges, we found a combination of phases where the transport speed, *ν*_1/2_, is enhanced by 7.68%. In Figure 2b, the enhancement of the time dependent occupation probability is shown for the chiral quantum walk. We note that optimization over only three edges already changes the transport speed by 5.92%, see Supplementary Information, Section S3.

Complex network theory has been used in abstract studies of quantum information science; see for example^30^,^31^. Here we turn to the theory of complex networks to determine if optimization procedures limited to small subsets of edges will generally lead to improved transport in larger and possibly randomly generated networks. We found a positive answer when testing the site-to-site transport between oppositely aligned nodes in the Watts-Strogatz model^32^. This family of small-world networks continuously connects a class of regular cyclic graphs to that of completely random networks (Erdős-Rényi models^33^) by changing the value of the rewiring probability.

**Figure 3:**
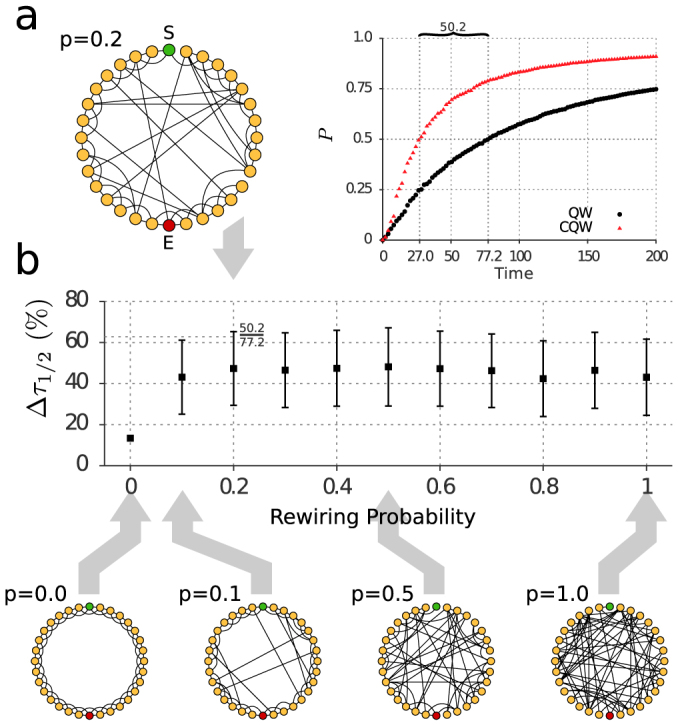
Transport enhancement of the chiral quantum walk is robust across randomly generated Watts-Strogatz networks. An example of this small-world network, with rewiring probability *p* = 0.2, is depicted in (a). The transfer probability *P* from site *S* to the sink connected to site *E* is plotted in a realization of the network. (b) shows the average enhancement of half arrival time (Δ*τ*_1/2_) for different values of *p*.

We numerically investigated graphs with 32 nodes, average degree four and range over rewiring probability *p* considering 200 different graph realizations for each value of *p*. An example with *p* = 0.2 is depicted in Figure 3a. Here the occupancy of a sink connected to site *E* is compared between the chiral walk and its achiral counterpart. The particle begins at site *S* and we perform the optimization of the phases only on edges connected to site *E*. In the case of the chiral quantum walk, the sink reaches half-occupancy in 54.8% less time on average.

## Discussion

In all the examples studied, we found that the effect that TRS breaking has on transport is non-trivially affected by the topology of the network. In this regard, a key observation is the following. If two Hamiltonians are related by on-site unitary transformations mapping |*n*〉 to 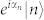, then the site-to-site transition probabilities will be identical. This fact provides a tool to reduce the effective space of phase parameters for controlling transport. In the Methods Section, we provide a more formal treatment of this symmetry of the site-to-site transition probabilities. For instance, we prove that the site-to-site transfer probability is insensitive to phases in tree graphs. For bipartite graphs the phases can have an effect, however, the dynamics still remains time-reversal symmetric.

A further consequence is that the sums of phases along a chosen orientation of a loop are the unique invariants under on-site unitary transformations. For example, placing phases on the edges of the triangle loop of the quantum switch is equivalent to placing the sum of them on just one edge. In a wide range of cases and particularly in all examples we considered, we found strong evidence of the robustness the effect has on transport. For instance, the examples in Figure 3b show that in the Watts-Strogatz model, the transport enhancement due to the time reversal asymmetry of the Hamiltonian is insensitive to changes of the rewiring probability *p* and the clustering coefficient measuring the density of triangles in the graph. Finally, additional calculations show that scale free networks such as the Barabási-Albert model^34^, show a similar transport enhancement, indicating robustness also with respect to the degree distribution.

This study pioneers the exploration of a new degree of freedom that allows for a significant improvement of control in the engineering of quantum transport. The fact that we were able to optimize and control transport by adjusting the phase on only a few edges inside a complex network and that the effect was relevant in a host of examples adds optimism to the robustness of this approach. Experimental demonstrations of the effects we predict are within reach of existing hardware, as outlined in the Supplementary Information, Section S2.

## Methods

### Analytical methods

#### Site-to-site transfer probability

The Markovian open-system dynamics of a continuous time chiral quantum walk is given by the Kossakowski-Lindblad equation^17^,^18^,^19^,^20^

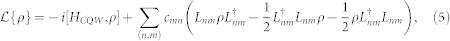
where the chiral Hamiltonian *H_CQW_* is defined in Eq. (2), and the Lindblad operators are given as *L_mn_* = |*m*〉〈*n*| with *c_nm_* ≥ 0. Transport from vertex |*S*〉 to vertex |*E*〉 during such dynamics is characterized by the site-to-site transfer probability (STP). In the unitary case (*c_nm_* = 0) it is given by 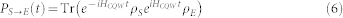
with *ρ_S_* = |*S*〉〈*S*| and *ρ_E_* = |*E*〉〈*E*|, while for the general Markovian case it is 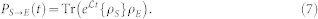


#### Time-reversal symmetry of the unitary achiral dynamics

In the setting of quantum walks, the time-reversal operator *T* acts as complex conjugation (with respect to the vertex basis)^24^: 
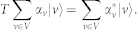


The antiunitarity of *T* and *T*^2^ = **1** implies that *T* = *T*. The time-reversal of a Hamiltonian *H* is given as *THT*( = *THT*). The 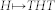 action is represented in parameter space by the replacement 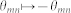 in Eq. (2). Thus exactly the achiral quantum walks are left invariant by this action. The STP's of *H* (*P_S_*_→*E*_(*t*)) and that of 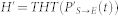 are related in the following way: 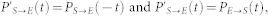
which can be verified using *Tρ_v_T* = *ρ_v_* and the cyclicity of the trace as follows: 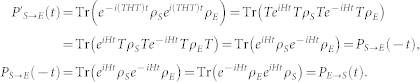


A crucial consequence of the above is that in the case of achiral quantum walks, the transition probabilities are the same at time *t* and −*t*, i.e. *P_S→E_*(*t*) = *P_S→E_*(−*t*), and directional biasing is prohibited *P_S→E_*(*t*) = *P_E→S_*(*t*). However, *H* ≠ *THT* does not necessarily imply that transition rates are asymmetric in time. This is because *THT* might be gauge-equivalent to *H*, as will be seen in the next section.

#### Gauge transformations

Formal gauge transformations, already introduced in the early work of Peierls^11^, are useful tools to study our models. Such a transformation is simply a local change of basis, i.e., a diagonal unitary 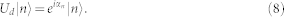
Here we collect a few of its properties and generalize them for the case of open systems with a Markovian bath. For us the starting point will be that it leaves the STP invariant. To prove this, let us first note that any unitary basis-change *U* would induce a transformation on the Lindblad superoperator 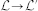 with 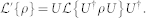


Using this and the invariance of localized states under diagonal unitaries (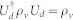), we arrive at 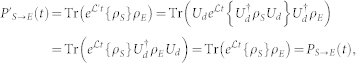
which proves the invariance of the STP under the gauge transformations defined by Eq. (8).

**Figure 4:**
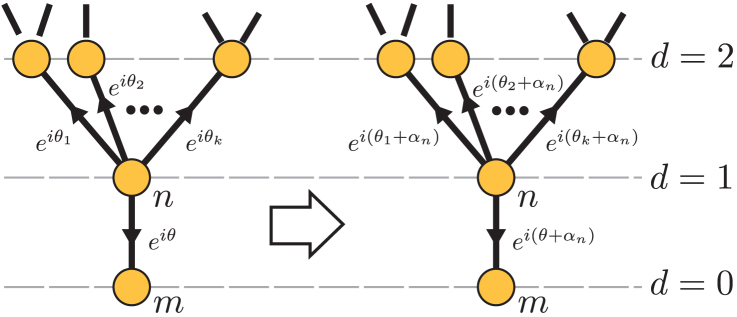
Effect of the gauge transformation 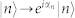 on vertex *n*. Phases on edges can be gauge-transformed without changing the transition amplitudes, as described in the text. Here we arrange the graph as a tree rooted at *d* = 0.

Under these diagonal transformations, the parameters of the quantum walk Hamiltonian transform as 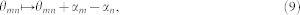
as illustrated in Figure 4. The incoherent part of the Lindblad equation (5) does not change since the Lindblad operators transform as 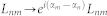 and these phases cancel in Eq. (5), since *L_nm_* and 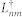 appear paired. Two important properties of the model now follow: (*i*) phases on tree graphs can be transformed out completely and (*ii*) the sum of phases along loops is invariant under gauge transformations.

The first property is illustrated in Figure 4. Let us take an arbitrary tree graph and pick a vertex *m* with only one neighbour. Redraw every other vertex on successive levels characterized by the distance *d* of the vertexes from the given vertex *m*. Note that the number of edges connecting two vertices, *d*, is by definition, unique in tree graphs. In such an arrangement only one edge emanates downwards from a given vertex on a line of *d* > 0 so Figure 4 represents the general neighbourhood of a vertex *n* having distance *d* = 1 from *m*. The indicated gauge transformation with *α_n_* = −*θ* removes the phase from the bottom edge. Then, one iterates the procedure for all vertices at level *d* = 2 and consecutively for all levels. In this way, all phases are removed. For the second property, pick an orientation on a loop of *N* vertexes and compute 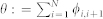, considering *ϕ_N_*_,*N*+1_ ≡ *ϕ_N_*_,1_. A gauge transformation 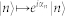, according to Eq. (9) leads to: 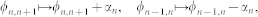
so the sum *θ* remains unaffected.

### Numerical methods

We used the Quantum Information Toolkit^35^. This is a software package for the Matlab programming language. The optimization procedure used in the FMO and the Watts-Strogatz examples rely on the *Interior-point Optimization* algorithm of the Matlab minimization tool kit. We start the optimization procedure several times from different randomly chosen points of the parameter space, to reach the global minimum of cost function with more certainty. Source code for all simulations done in this work is available upon request.

## Author Contributions

Z.Z., M.F., Z.K., J.W., B.L. and J.B. contributed to the development of the present study and to the drafting of the manuscript. B.L. conceived the experimental proposal. All authors assisted in the writing of the manuscript.

## Supplementary Material

Supplementary Information Supplementary Information
