# Supplementary material for: Quantum Transport Enhancement by Time-Reversal Symmetry Breaking
Source: Sci Rep. 2013 Aug 6;3:2361. doi: 10.1038/srep02361 (PMC3734483; doi:10.1038/srep02361)
Supplement: Supplementary Information [file srep02361-s1.pdf]

Supplementary Information for
Quantum Transport Enhancement by Time Reversal Symmetry Breaking

Zoltán Zimborás[1,2], Mauro Faccin[1], Zoltán Kádár[1], James Whitfield[1,3], Ben Lanyon[4], and Jacob Biamonte[5,1,*]

[1]*Institute for Scientific Interchange, Via Alassio 11/c, 10126 Torino, Italy*
[2]*Department of Theoretical Physics, University of the Basque Country UPV/EHU, P.O. Box 644, E-48080 Bilbao, Spain*
[3]*Vienna Center For Quantum Science and Technology, Boltzmanngasse 5 1090 Vienna, Austria*
[4]*Institut für Quantenoptik und Quanteninformation, Otto-Hittmair-Platz 21a 6020 Innsbruck, Austria*
[5]*Centre for Quantum Technologies, National University of Singapore, Block S15, 3 Science Drive 2, Singapore 117543*
[*]*To whom correspondence can be addressed; E-mail:* `jacob.biamonte@qubit.org`

S1 Analytic examples for enhancement, suppression and direction

Using the example of the regular polygon (that has $J_{mn} \equiv 1$ for all edges) we will explain how enhancement, suppression and direction of transport can be influenced by the phases in chiral quantum walks. Typically we need to solve the eigenvalue problem of the Hamiltonian $H|\tilde{j}\rangle = E_j|\tilde{j}\rangle$ (the tilde denotes energy eigenstates) to compute

$$P_{S\to E}(t) = \left| \sum_{\tilde{j}}^{N} \langle E|\tilde{j}\rangle\langle\tilde{j}|S\rangle e^{-iE_j t} \right|^2$$

For a regular homogeneous polygon ($H = \sum_{n=1}^{N} e^{i\varphi}|n\rangle\langle n+1| + h.c.$ with cyclic boundary condition) the eigenvectors and eigenvalues are found from the Fourier Transform

$$|\tilde{k}\rangle = \frac{1}{\sqrt{N}} \sum_{n=1}^{N} e^{-\frac{2\pi i k n}{N}} |n\rangle \ , \quad E_k(\varphi) = 2\cos\left(\frac{2\pi k}{N} - \varphi\right) \ ,$$

The STP from site S to E then reads

$$P_{S\to E}(t,\varphi) = \left| \frac{1}{N} \sum_{k=0}^{N-1} e^{-i\left(2t\cos(\frac{2\pi k}{N} - \varphi) + \frac{2\pi k(E-S)}{N}\right)} \right|^2 \tag{S1}$$

We now list several features

- Direction and enhancement

  In the case of time symmetric quantum walks, for the homogeneous polygon $P_{S\to E} = P_{S\to N-E+2S}$. This implies that if the number of edges is odd, the STP is at most $1/2$, since there is always a vertex different from E at which the probability is identical. The chiral case breaks this symmetry. In particular, for the regular triangle one can reach $P_{1\to 2}(t) = 1$; one can find values of θ analytically by equating the phases of the three exponentials in the STP modulo 2π and solve the two linear equations for t and θ. Enhancement and direction is indicated in Fig. S1, where a triangle graph is shown with inhomogeneous coupling.

- Complete suppression of transport

  Complete suppression of transport is possible in loops with an even number of sites. They belong to the set of *bipartite* graphs, whose vertices can be partitioned into two sets such that edges connect only members from different subsets. Directing transport in bipartite graphs is forbidden. This can be easily shown by noting that the gauge transformation $|n\rangle \mapsto -|n\rangle$ for all members of one subset has the effect of $H \mapsto -H$, which is precisely the time reversal transformation. Hence

$$P_{S\to E}(t) = P_{S\to E}(-t) = \mathrm{Tr}(e^{iHt}\rho_S e^{-iHt}\rho_E) = \mathrm{Tr}(e^{-iHt}\rho_E e^{iHt}\rho_S) = P_{E\to S}(t) \ .$$

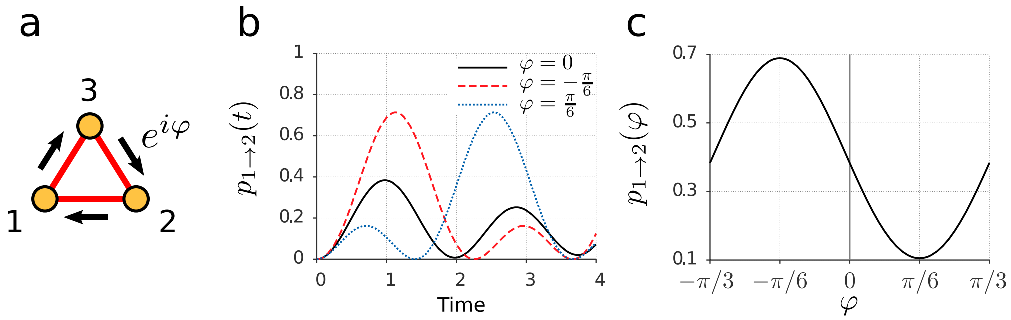

Figure S1: (**A**) Triangle with inhomogeneous coupling and chiral phases. (**B**) Illustrates the quantity $P_{1\to2}(t)$ for the triangle graph with inhomogeneous coupling $J_{12} = 1, J_{23} = 1.3, J_{13} = 0.5$. Directing the phases of the chiral walker effects the transport, in particular, there is enhancement for $\theta = 3\varphi = \pi/2$ in both arrival time and maximal probability. (**C**) The probability varies as a function of the chiral angle at a given time near the first maximum.

Even chiral loops suppress transport as $\theta = \pi$ results in $p_{1\to N/2+1}(t) = 0$ for all times t. This can again be shown by writing down the formula (S1) and separating it into two sums, one for k being even and the other for k odd. The simplest even polygon is the quadrilateral that can be used as a building block for the realization of transport suppressing topologies.

Robustness of chiral transport enhancement of the triangle chain In the triangle chain discussed in the main text, the half arrival time $\tau_{1/2}$ depends linearly on the size of the system, see Figure S2. This fact is a characteristic of quantum walks in linear chains and continues to hold for the chiral triangle chain.

S2 Experimental proposal

In this section we outline a proposal to simulate chiral quantum walks in a system of ultra-cold trapped atomic ions. The proposal can be realized with currently available techniques and technology. Trapped ion systems have previously been used to investigate non-chiral quantum walks [7], where the motional state was used to encode the walk. In contrast, in our proposal both chiral and non-chiral walks are encoded into the electronic state of the ions. The walk dynamics is generated by ion-ion interactions mediated by joint vibrational modes of the ion string. These interactions can be driven by laser-induced optical dipole forces, for example, and can be implemented with high quality as shown in several recent works [8,9]. In the text below we first introduce the 3-ion system that the experiment requires. Then we explain how to engineer the quantum walk Hamiltonians that we wish to investigate. Next, we calculate the chiral and non-chiral phenomena that can be generated by these Hamiltonians. Finally, we outline the experimental procedure and note some important experimental considerations.

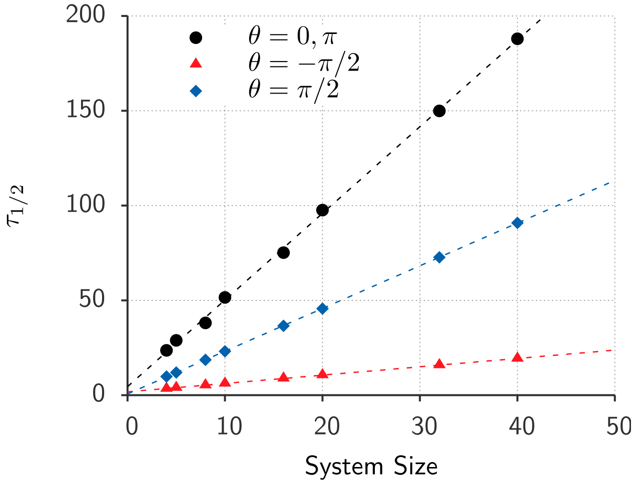

Figure S2: Half arrival time scaling. Dependence of the half arrival time, $\tau_{1/2}$, on the length of the triangle chain shown in Figure 2A for different values of the phases. Both chiral ($\theta \neq 0$) and achiral ($\theta = 0$) cases exhibit linear scaling behaviour. The slope can be tuned by changing the value of the phase parameter.

Implementation in trapped ions We consider a string of three ions in a linear ion crystal, which can be achieved with standard linear-Paul traps. A long-lived electronic transition inside each ion encodes a two-level spin. The mapping could be that spin-down is represented by an ion in the ground state, while spin-up is represented by an ion in the excited state of this transition, for example. The 8 possible logical states of this system then become $|\downarrow,\downarrow,\downarrow\rangle, |\downarrow,\downarrow,\uparrow\rangle, |\downarrow,\uparrow,\downarrow\rangle, ..., |\uparrow,\uparrow,\uparrow\rangle$. In our proposal a subset of these states represents the sites of the quantum walk, as will be described. The dynamics of any quantum walk between these states require the ability to turn on interactions between the ionic spins. Effective ion-ion interactions can be achieved by off-resonantly coupling the electronic states in which the spin is encoded to one or more common vibrational modes of the string. Such a spin-dependant force can be implemented using a laser field with two symmetrically tuned frequencies at $\omega_s \pm \mu$ with $\mu \ll \omega_s$. If the symmetric detuning is sufficiently far from all motional side-bands so that the generation of phonons can be adiabatically eliminated [10], then a pure spin-spin interaction is generated of the form:

$$H(\phi) = \sum_{i<j} J_{ij} \sigma_\phi^i \sigma_\phi^j \,, \tag{S2}$$

where

$$\sigma_\phi^i = \cos\phi \, \sigma_x^i + \sin\phi \sigma_y^i$$
$$J_{ij} = \frac{\Omega^2 \eta^2 \omega}{\Delta^2 - \omega^2} b_i b_j \tag{S3}$$

Here Ω describes the laser coupling strength to the electronic transition, η_i is the Lamb-Dicke parameter describing the coupling strength between the laser and the motional side-band for a

single ion. An experimentally tunable phase ϕ is set by the phase difference of the two driving laser fields. Finally, b_i describes how strongly ion i couples to motional mode ω. The increasing difficulty of generating spin-spin interactions with more ions in a string is incorporated in b_i.

In this proposal we will simultaneously use two of the normal modes along the axis of the string, called the centre-of-mass (COM) and breathing (Br) modes, to mediate spin-spin interactions. These modes are typically well-spaced in frequency and can therefore can be individually addressed. The coupling vectors b_i for the axial COM and breathing are $1/\sqrt{3} \times [1, 1, 1]$ and $1/\sqrt{2} \times [1, 0, -1]$, respectively. Consequently, the contributions to the spin-spin interaction Hamiltonian of these modes are:

$$H_{COM}(\phi_1) = J_{COM}\,(\sigma^1_{\phi_1}\sigma^2_{\phi_1} + \sigma^2_{\phi_1}\sigma^3_{\phi_1} + \sigma^1_{\phi_1}\sigma^3_{\phi_1}) \tag{S4}$$

$$H_{Br}(\phi_2) = J_{Br}\,\sigma^1_{\phi_2}\sigma^3_{\phi_2} \tag{S5}$$

where

$$J_i = \frac{\Omega_i^2 \eta_i^2 \omega_i}{\Delta_i^2 - \omega_i^2} \tag{S6}$$

determines the coupling strength of the spin-spin interaction due to vibrational mode i. This equation allows for separate pairs of light fields to drive each mode simultaneously, which can therefore have different coupling strengths and detunings. This is required because we wish to be able to precisely control the relative strengths A_i, which can be achieve by changing the relative strengths of the laser fields. Separate pairs of light fields are also necessary since we require the ability to individually set the phase ϕ_i determined by the phase difference between the laser fields in each pair.

Ideal experimental behavior of the proposed walks Now that we have established the Hamiltonians to drive the quantum walks, given by equations (S4) and (S5), we will examine the walk dynamics that we wish to explore. A key observation is that the operators $J\,\sigma^m_\phi\sigma^n_\phi$ $(m, n \in \{1, 2, 3\}, m \neq n)$ leaves invariant the subspace spanned by the following four spin states

$$|1\rangle \equiv |\uparrow,\downarrow,\downarrow\rangle, \quad |2\rangle \equiv |\downarrow,\uparrow,\downarrow\rangle, \quad |3\rangle \equiv |\downarrow,\downarrow,\uparrow\rangle, \quad |4\rangle \equiv |\uparrow,\uparrow,\uparrow\rangle\,.$$

In order to see this, consider the following decomposition $(m, n \in \{1, 2, 3\},\ m \neq n)$:

$$\sigma^m_\phi\sigma^n_\phi = \left(\cos\phi\sigma^m_x + \sin\phi\sigma^m_y\right)\left(\cos\phi\sigma^n_x + \sin\phi\sigma^n_y\right) \tag{S7}$$

in terms of the creation and annihilation operators $\sigma^m_\pm = \sigma^m_x \pm i\sigma^m_y$. It follows that:

$$\sigma^m_\phi\sigma^n_\phi = e^{-2i\phi}\sigma^m_+\sigma^n_+ + e^{2i\phi}\sigma^m_-\sigma^n_- + \sigma^m_+\sigma^n_- + \sigma^m_-\sigma^n_+ \tag{S8}$$

Here $|n\rangle$ and $|m\rangle$ are coupled by the real strength J, whereas $|4\rangle$ is coupled to the remaining site by the same strength multiplied with the phase $e^{-2i\phi}$. Now, notice that the modes H_{COM} and H_{Br}

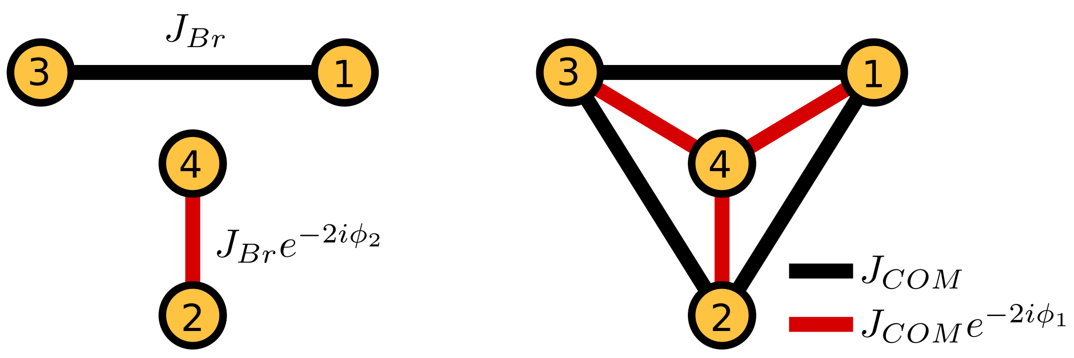

Figure S3: Networks describing the modes H_{Br} (left) and H_{COM} (right).

are built from operators of the above form. Using these models we can realize quantum walks on the four sites given above. Now one determines the parameters of the quantum walk Hamiltonians:

$$H^{(k)} = \sum_k \sum_{n,m=1}^{4} J_{nm}^{(k)} |n\rangle\langle m| \quad \text{with} \quad J_{nm}^{(k)} = \overline{J}_{mn}^{(k)} \, ,$$

where the index k refers to the two different modes $H_{COM}(\phi_1)$ and $H_{Br}(\phi_2)$. Figure S3 shows the general situation.

The values to be used for the experiment are given in the following table:

	J_{COM}	J_{Br}	ϕ_1	ϕ_2
H_{CQW_1}	2	-3	$\pi/2$	$0.304\,\pi$
H_{CQW_2}	2	-3	$\pi/2$	$-0.304\,\pi$
H_{QW}	-2	1	$\pi/2$	0

The parameter ϕ_2 is determined by $\cos 2\phi_2 = -1/3$. Since the effect ϕ_1 is just a minus sign in H_{COM} for the edges incident to $|4\rangle$, we see that H_{CQW_1} and H_{CQW_2} are the images of each other under TRS transformation. One can also check that H_{QW} is the time-reversal symmetric counterpart of the chiral walks in that all its couplings are real and equal to the absolute values of the chiral walks after we relabel sites $|2\rangle$ and $|4\rangle$. Computer simulation (Figure S4) shows the effect of TRS breaking, since $P_{1\to2}(t) \neq P_{2\to1}(t)$, enhancement and suppression of transport as the first maxima of H_{CQW_2} and H_{CQW_1} are of considerably larger (smaller) magnitude, respectively, than that of the corresponding symmetric walker. When we compute $P_{2\to1}(t)$, the roles of the two chiral walks are exchanged due to the symmetry of the model.

Experimental details As a specific example we consider using three $^{40}Ca^{+}$ ions in a standard linear Paul trap. This atomic species is used by several groups around the world and can be pre-

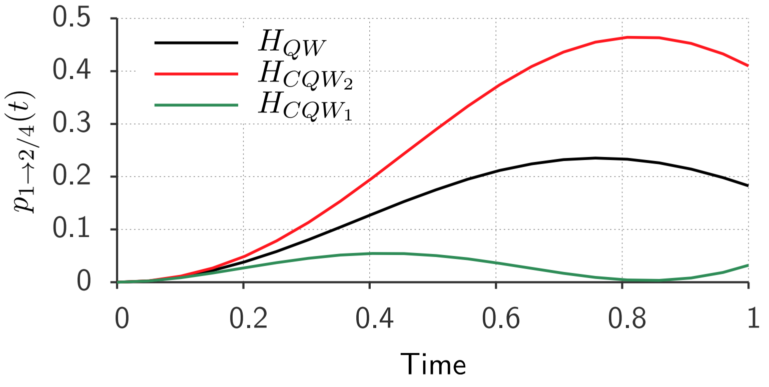

Figure S4: The effect of the chiral phase parameter on the occupation probability. The plot shows the chiral walkers at site 2 and the achiral one at site 4, all being initially at site 1. By this arrangement the real coupling strengths of the corresponding Hamiltonians are the same.

cisely manipulated in the way that we require. Spins can be encoded into a metastable electric quadrupole transition, the excited state of which has a lifetime of approximately 1 second which is much longer than the times required for quantum operations. Spin operations can be driven using a 729nm laser, spin-spin interactions can be implemented using a bichromatic light field with symmetrical detuned side-bands around the electronic transition [11]. We note that implementation of our proposal using another atomic species with a hyperfine transition, for example, would also be possible.

Figure S5 shows one possibility allowing simultaneous realisation of the Hamiltonians given in equations (S4) and (S5). Two pairs of bichromatic fields are turned on, on pair tuned close to the axial COM and another to the axial breathing mode. We choose to use the axial modes, since they are well-spaced out in frequency, thereby enabling each bichromatic light field to be simultaneously close enough to its nearest vibrational mode such that the effect of that mode completely dominates the spin-spin interaction and sufficiently far away such that the adiabatic approximation still applies.

The criterion to generate a pure spin-spin interaction with each bichromatic field is $|\omega_m - \mu| \gg \eta_m\omega_m$, i.e. the detuning from all side-bands is much greater than the coupling strength on those modes. Using the detunings shown in Figure S5, and Ω=$2\pi \times 100KHz$ we obtain ratios $|\omega_m - \mu|/\eta_m\omega_m$ of 21 and 14 for the COM and breathing bichromats, respectively. In this regime, errors due to the generation of phonons are extremely small. Regarding the issue of off-resonant coupling to unwanted modes: straightforward calculations using equation (S6) and the frequencies shown in figure S5 show that in both cases the size of the far-offresonant spin-spin coupling, due to bichromatic field 2 on the COM for example, is more than one order of magnitude less than the desired couplings in every case. This could be further reduced by increasing the frequency separation between axial modes by increasing the confining potential in this direction, for example.

An important experimental consideration is the requirement to maintain a fixed phase relationship between the two pairs of light fields generating the walk Hamiltonians. For example, this can be achieved by generating them in the same acousto-optic modulator (AOM) and thereby keeping their paths common mode, between the point of generation and the point of interaction with the ions. The maximum frequency splitting is approximately 3.4 MHz, which will result in some angular divergence of the different frequencies at the output facet of the AOM crystal, which can be compensated with linear optics allowing coupling into an optical fibre for transport to the ion trap itself.

The experiment would proceed as follows. Firstly, standard methods of doppler cooling, resolved side-band cooling on the axial COM and breathing modes, and optical pumping prepares the three ion string into an ultra low entropy state and the initial spin state $|\downarrow,\downarrow,\downarrow\rangle$. Next, a standard combination of single-ion focused and three-ion focused beams is used to prepare the initial state $|\uparrow,\downarrow,\downarrow\rangle$. The bichromatic light fields which implement the desired walk dynamics are then turned on for a fixed period of time. Finally the state of each encoded spin (up or down) is measured individually using standard fluorescent detection techniques and a CCD camera. Experiments are repeated multiple times from which estimates of the probability for finding spins in all configurations can be deduced.

S3 Fenna-Matthews-Olson complex

The Fenna-Matthews-Olson (FMO) complex is a well studied light harvesting system in green sulphur bacteria. The interest in FMO resides in the high efficiency shown in the transport of energy from the antenna to the reaction centre. The faithful description and simulation of the FMO complex has been the highlight of recent research.

The system is composed of three subunits, each made of seven BChl-a molecules embedded in a protein scaffold. We restrict our study to the simulation of the exciton transport in a single unit. To simulate the evolution of the system in the one-excitation manifold, we use the following Liouville equation:

$$\dot{\rho} = \mathcal{L}[\rho] = -i[H,\rho] + \phi\hat{L}_\phi\{\rho\} + \gamma\hat{L}_\gamma\{\rho\} + \tau\hat{L}_\tau\{\rho\} \tag{S9}$$

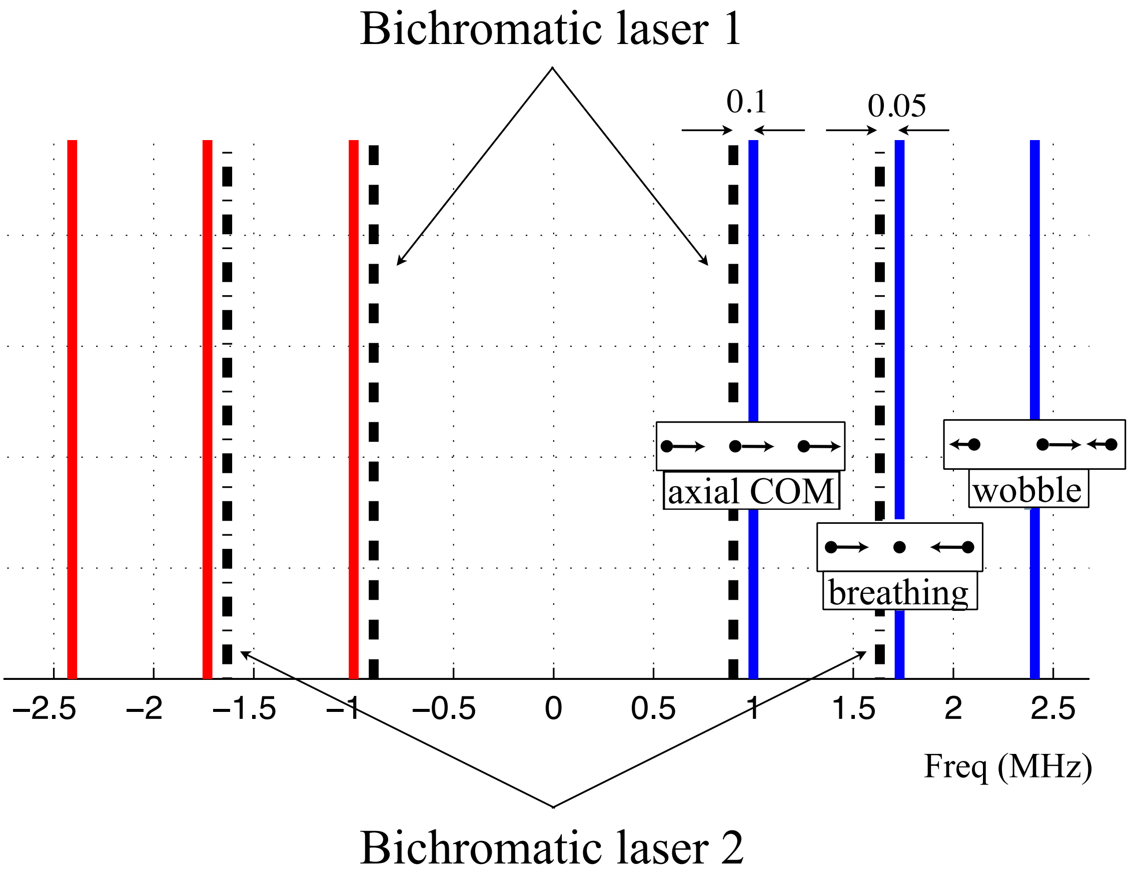

Figure S5: Mode frequency spectrum of a linear three ion crystal of $^{40}Ca^{+}$ in a typical harmonic trap with radial COM (not shown) and axial COM frequencies of 5 MHz and 1MHz, respectively. Two bichromatic laser fields, each symmetrically detuned from the axial COM (by 100KHz) and breathing modes (by 50KHz), drive interactions between spins encoded into an electronic transition of each ion. The phase difference between the spectral components in bichromatic field 1 (2) determines the phase ϕ in the Hamiltonian given in equation (S4) ((S5)).

where the Lindblad super-operators are defined as:

$$\hat{L}_{\phi}\{\rho\} \equiv \sum_{n} L_{\phi,n}\rho L_{\phi,n}^{\dagger} - \frac{1}{2}\left(L_{\phi,n}^{\dagger}L_{\phi,n}\rho + \rho L_{\phi,n}^{\dagger}L_{\phi,n}\right) \qquad L_{\phi,n} = |n\rangle\langle n| \tag{S10}$$

$$\hat{L}_{\gamma}\{\rho\} \equiv \sum_{n} L_{\gamma,n}\rho L_{\gamma,n}^{\dagger} - \frac{1}{2}\left(L_{\gamma,n}^{\dagger}L_{\gamma,n}\rho + \rho L_{\gamma,n}^{\dagger}L_{\gamma,n}\right) \qquad L_{\gamma,n} = |d\rangle\langle n| \tag{S11}$$

$$\hat{L}_{\tau}\{\rho\} \equiv \sum_{n\in T} L_{\tau,n}\rho L_{\tau,n}^{\dagger} - \frac{1}{2}\left(L_{\tau,n}^{\dagger}L_{\tau,n}\rho + \rho L_{\tau,n}^{\dagger}L_{\tau,n}\right) \qquad L_{\tau,n} = |\tau\rangle\langle n| \tag{S12}$$

where the sums are over the site basis and T is the set of sites connected to the reaction centre (in this case only site three). The coherent part of the evolution is described using a time reversal symmetric Hamiltonian from the literature [12]. In (S9) the non-coherent terms describe the coupling of the system with a thermal bath, dephasing, Eq. (S10), and recombination, Eq. (S11), and the effect of the excitation trapping at the site connected to the reaction centre, Eq. (S12). The thermal bath, for the present paper, is considered as a set of harmonic oscillators coupled to the system of interest as in [3,13].

The initial state is set on site one and the reaction centre is connected as an energy sink to site three. In the simulation we use a dephasing rate of $\phi = 9.0$ ps^{-1} (which corresponds to 295K), the recombination rate is $\gamma = 1$ ns^{-1} and trapping rate from site three to the reaction centre is $\tau = 1.0$ ps^{-1}.

The optimization of the phases was found to be robust with respect to phase changes. The resulting phases are reported in Table S1.

S4 Small-world networks

The Watts-Strogatz model [14] gives a constructive algorithm for building a network with small-world characteristics, starting from a regular lattice. The latter is defined as a periodic chain of N nodes where each node is connected to k neighbours ($k/2$ to the left and $k/2$ to the right). The final small-world network is obtained by taking all neighbouring edges at each node and rewiring all the edges toward the node left with a probability p. The limit for $p \to 1$ of the Watts-Strogatz model is the Erdős-Rényi random model with fixed number of edges (restricted to connected graphs).

In our case, we set $N = 32$ and $k = 4$ and choose only connected graphs as we are concerned in comparing transport on networks of the same sites. The initial state is on site S while the sink is a external site connected to site E. Site E latter is placed on the opposite side of the initial circle. The absorption rate of the sink is $r = 1.0$. The evolution is described by the coherent Hamiltonian, which, in this case, corresponds to the connectivity matrix of the network. We define $\tau_{1/2}^{QW}$ the time needed by the normal quantum walker to reach the probability $p_{sink}(t) = 1/2$. We add phases to the edges neighbouring node E and optimize them in order to improve the probability

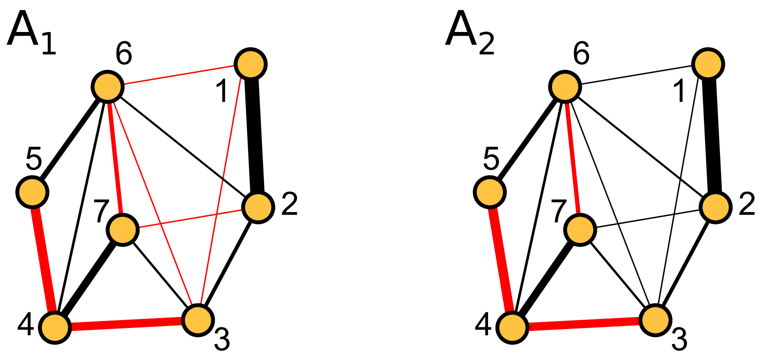

Edges	A_1	variance	A_2	variance
3-4	$1.31484899\,\pi$	$4.33 \cdot 10^6\pi$	$1.58371001\,\pi$	$4.86 \cdot 10^6\pi$
4-5	$1.66997830\,\pi$	$5.20 \cdot 10^6\pi$	$1.39551582\,\pi$	$6.60 \cdot 10^6\pi$
6-7	$1.8406103\,\pi$	$1.25 \cdot 10^5\pi$	$0.1338368\,\pi$	$1.72 \cdot 10^5\pi$
2-7	$1.2949616\,\pi$	$2.80 \cdot 10^5\pi$		
1-6	$1.67543320\,\pi$	$5.36 \cdot 10^6\pi$		
1-3	$0.04222214\,\pi$	$4.36 \cdot 10^6\pi$		
3-6	$0.8761298\,\pi$	$1.03 \cdot 10^5\pi$		

Table S1: Transport enhancement in the FMO complex. Both for A_1 and A_2, complex phases are applied to the red edges. The results of a simultaneous optimization procedure are listed in the table.

of being trapped in the target site at time $\tau_{1/2}^{QW}$. The non-coherent part (trapping) is described by a Lindbladian super-operator. We repeat the evolution for 200 different realizations of the graph for each value of p.

1. Kossakowski, A. On quantum statistical mechanics of non-Hamiltonian systems. *Rep. Math. Phys* **3**, 247 (1972).

2. Lindblad, G. On the generators of quantum dynamical semigroups. *Commun. Math. Phys* **48**, 119 (1975).

3. Breuer, H.-P. & Petruccione, F. *The theory of open quantum systems* (Oxford University Press, 2002).

4. Whitfield, J. D., Rodriguez-Rosario, C. A. & Aspuru-Guzik, A. Quantum stochastic walks: A generalization of classical random walks and quantum walks. *Phys. Rev. A* **81**, 022323 (2010).

5. Wigner, E. P. *Group Theory and its Application to the Quantum Mechanics of Atomic Spectra* (New York: Academic Press, 1959). Translation by J. J. Griffin of 1931, Gruppentheorie und ihre Anwendungen auf die Quantenmechanik der Atomspektren, Vieweg Verlag, Braunschweig.

6. Peierls, R. On the Theory of Diamagnetism of Conduction Electrons. *Z. Phys.* **80**,763791 1933.

7. Zähringer, F. *et al.* Realization of a quantum walk with one and two trapped ions. *Phys. Rev. Lett.* **104**, 100503 (2010).

8. Lanyon, B. P. *et al.* Universal digital quantum simulation with trapped ions. *Science* **334**, 57–61 (2011).

9. Kim, K. *et al.* Quantum simulation of frustrated Ising spins with trapped ions. *Nature* **465**, 590–593 (2010).

10. Kim, K. *et al.* Quantum simulation of the transverse ising model with trapped ions. *New J. Phys.* **13**, 105003 (2011).

11. Kirchmair, G. *et al.* Deterministic entanglement of ions in thermal states of motion. *New Journal of Physics* **11**, 023002 (2009).

12. Adolphs, J. & Renger, T. How proteins trigger excitation energy transfer in the FMO complex of green sulfur bacteria. *Biophys. J.* **91**, 2778–2797 (2006).

13. Mohseni, M., Rebentrost, P., Lloyd, S. & Aspuru-Guzik, A. Environment-assisted quantum walks in photosynthetic energy transfer. *J. Chem. Phys.* **129**, 174106 (2008).

14. Watts, D. J. & Strogatz, S. H. Collective dynamics of ‘small-world’ networks. *Nature* **393**, 409–410 (1998).
